# Point-of-care airway US: a valuable tool in the management of occult over the cuff bleeding and secretions

**DOI:** 10.1186/s13089-022-00300-7

**Published:** 2023-02-02

**Authors:** Amedeo Bianchini, Cristina Felicani, Elena Zangheri, Matteo Bianchin, Antonio Siniscalchi

**Affiliations:** 1 Post-Surgical and Transplant Intensive Care Unit, Department of Digestive, Hepatic and Endocrine-Metabolic Diseases, IRCCS Azienda Ospedaliero-Universitaria di Bologna, Via Giuseppe Massarenti, 9, Bologna, Italy; 2 UOC Medicina ad Indirizzo Metabolico Nutrizionale. Policlinico di Modena, AOU Modena, Via del Pozzo, 71, Modena, Italy; 3 Department of Medical and Surgical Sciences (DIMEC), University of Bologna, Via Giuseppe Massarenti, 9, Bologna, Italy; 4 Anesthesiology and pain therapy, Sant’Orsola-Malpighi Hospital, University of Bologna, Via Giuseppe Massarenti, 9, Bologna, Italy

**Letter to the editor**

During upper airway instrumentation to secure the airway or surgical manipulation above a secured airway, occult blood or secretions might accumulate over the cuff of an endotracheal/tracheostomy tube.

This can happen during maxillofacial/ENT surgery or esophagogastroduodenoscopy (EGDS), after a difficult intubation, a nasal intubation or a tracheostomy.

Pooled blood and secretions over the cuff are a known risk factor for ventilator-associated pneumonia (VAP) [1]. Moreover, airway obstruction due to a large clot can lead to respiratory failure while inhalation of small clots or uncoagulated blood can cause obstruction of the distal airways and subsequently resorptive atelectasis.

When blood or secretions accumulate over the cuff, their direct and indirect visualization (FBS, DL, VL) can be hampered [4].

Airway ultrasound (US) could be a valuable tool in the management of this clinical situation, but its role has not yet been clearly identified and scientific knowledge on this topic is currently limited [2, 3]. We propose some possible US applications based on our clinical experience.

In normal conditions, the air–mucosal interface completely reflects the US beam and impedes visualization of the posterior structures, but when air is replaced by fluid an US acoustic window is generated. Tissue–blood or tissue–fluid interfaces are easily crossed by the US beam and allow visualization of the tracheal lumen content and the posterior tracheal wall.

In these conditions it is possible to scan the content of the tracheal lumen using a longitudinal or transverse probe orientation with a high frequency probe. With such technique it is thus possible to visualize the tracheal lumen content and distinguish between secretions, blood, or clots thanks to their different echogenicity (Fig. 1).

**Fig. 1:**
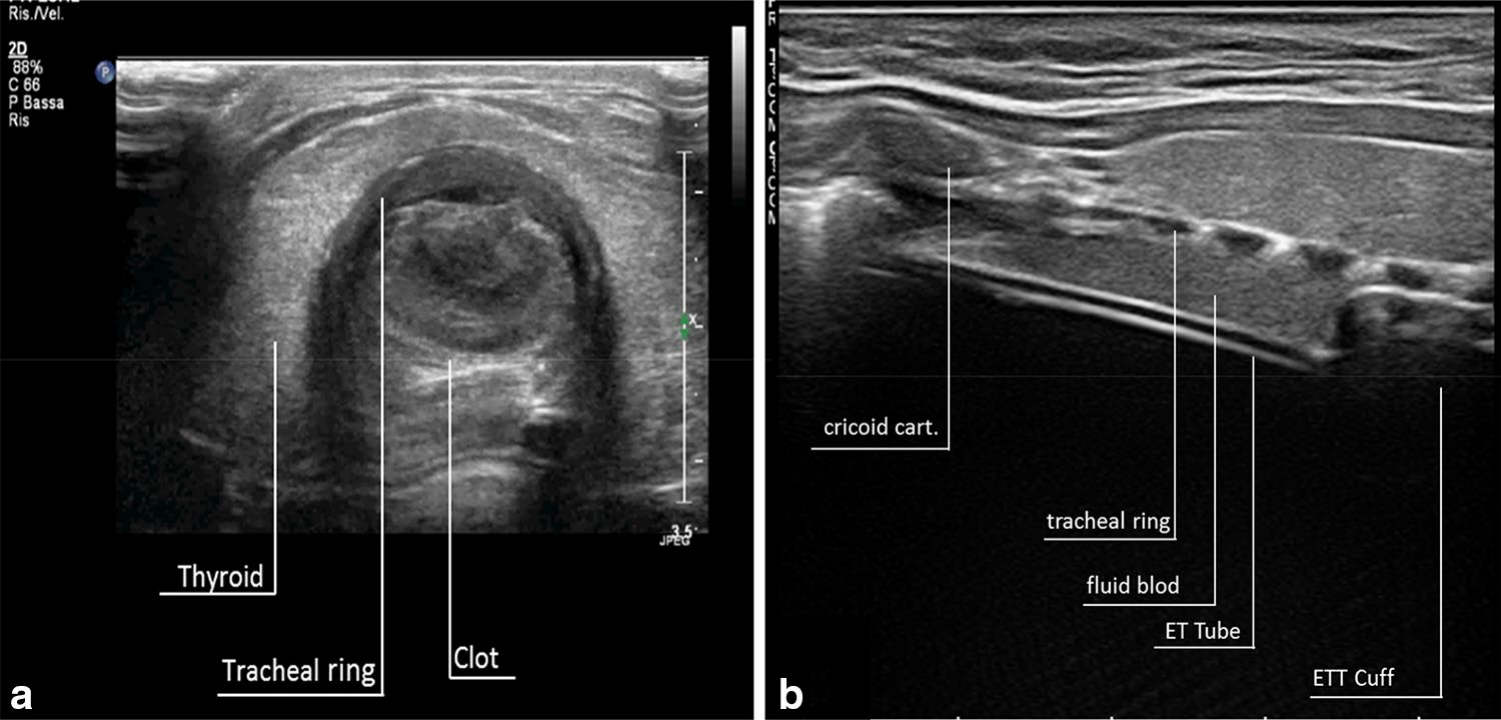
**a** Large clot inside the trachea. It appears as a slightly inhomogeneous echo-structure consisting of hyperechoic and hypoechoic areas. **b** Uncoagulated blood over the cuff after difficult intubation

In our opinion there are at least three common clinical situations in which biologic material might accumulate over the cuff and therefore this technique could foster a safer airway management.

In the first place, after ENT/maxillofacial surgery, which are high risk situations for occult over the cuff blood or secretions, excluding their presence could help the clinician proceed to a safer extubation.

Secondly, after invasive airway procedures (e.g., tracheostomy, nasal intubation or unpredicted difficult airway with multiple intubation attempts), upper airway ultrasound could help rule out once again occult accumulations of blood, secretions, or blood clots. This could prove particularly useful in the rare situation in which a blood clot forms over the cuff: if undetected, whether a further complication, such as cuff rupture or device dislocation, should ensue, it could prove fatal.

In the third place, this technique could prove useful in patients at high risk for gastric material accumulation over the cuff [5], such as patients with gastrointestinal sub-occlusion or after EGDS.

Across the spectrum of all these situations, if VAP prevention is considered, US evaluation of pooled blood or secretion could guide thorough aspiration before tracheostomy procedures or extubation.

The main limitations of this technique remain inter-operator variability and suboptimal ultrasound windows (e.g., subcutaneous emphysema, unfavorable neck anatomy).

In conclusion, airway ultrasound is a rapidly developing field and it could be a valuable tool in the management of occult over the cuff bleeding and secretions.

## Data Availability

Not applicable.

## Abbreviations

VAP: Ventilator-associated pneumonia
US: Ultrasound
EGDS: Esophagogastroduodenoscopy
FBS: Flexible bronchoscopy
DL: Direct laryngoscopy
VL: Video laryngoscopy
